# Virtual reality for assessment in undergraduate nursing and medical education – a systematic review

**DOI:** 10.1186/s12909-025-06867-8

**Published:** 2025-02-22

**Authors:** Andrea N. Neher, Florian Bühlmann, Martin Müller, Christoph Berendonk, Thomas C. Sauter, Tanja Birrenbach

**Affiliations:** 1 Department of Emergency Medicine, Inselspital, Bern University Hospital, University of Bern, Rosenbühlgasse 27, Bern, CH-3010 Switzerland; 2 Graduate School for Health Sciences, University of Bern, Bern, Switzerland; 3 Institute for Medical Education, University of Bern, Bern, Switzerland

**Keywords:** Medical education, Virtual reality, Performance assessment, Nursing

## Abstract

**Background:**

Virtual reality (VR) is increasingly used in healthcare education, offering immersive training experiences that are as effective as conventional methods, with benefits like cost-effectiveness, replicating complex scenarios, and reduced need for physical resources. However, the use of VR as an assessment tool is still emerging, particularly in nursing and medical education. The aim of this systematic review was to examine how immersive VR is used as an assessment tool for nursing and medical students.

**Methods:**

Embase, PubMed, PsycINFO, Cochrane, CINAHL, and ERIC were searched for articles that assessed nursing and/or medical students using immersive/HMD VR. The data was extracted, and content analysis was performed.

**Results:**

Twenty-six studies met the inclusion criteria, investigating VR assessments in various settings mostly emergencies. Assessments focused on core competencies Patient Care such as first triage, Interpersonal and Communication Skills (e.g., interprofessional communication), and Medical Knowledge (e.g., about coma), utilizing a range of assessment methods from *knowledge* to *performance* levels. VR was used either as an automated or supporting assessment tool. Practical considerations in VR implementation were also examined, such as hardware and software.

**Conclusion:**

The use of VR in medical education assessment shows promise, particularly for emergency scenarios and performance-based tasks related to core competencies such as Patient Care, Interpersonal and Communication Skills, and Medical Knowledge. While this technology offers opportunities to automate assessments and reduce examiner workload, challenges related to software, costs, and feasibility must be addressed. Additionally, aligning learning objectives, teaching methods, and VR assessments through constructive alignment is essential to ensure effective implementation as both a teaching and evaluation tool.

**Supplementary Information:**

The online version contains supplementary material available at 10.1186/s12909-025-06867-8.

## Introduction

In health care education, virtual reality (VR) has become widely available. It refers to an entirely virtual experience that encompasses everything one sees, moves, or interacts with. It exists in multiple forms, ranging from screen-based applications to highly immersive systems. These varying levels of immersion seem to have an impact on learning outcomes and should therefore be examined separately [1, 2]. In this study, we focus specifically on immersive VR, delivered through head-mounted displays (HMDs), to achieve the highest possible level of realism and immersion.

The training of medical students with immersive VR has been well-researched and has several advantages and disadvantages. Training covers everything from knowledge [3] and basic skills [4] to complex procedures [5], and full clinical scenarios [6]. Reviews suggest that VR is at least as effective as conventional training methods, particularly in skills training and knowledge [7, 8]. Compared to traditional methods, VR can be more cost-effective [9]; it requires less space and fewer additional resources, such as standardized patients or simulation manikins, medical supplies, or even supervisors. Situations that are difficult to recreate in real life, such as emergency scenarios [10, 11] can be replicated with VR without posing safety risks to patients and can be used location-independently [12]. However, users must first become familiar with VR technology before they can effectively operate it [13]. Additionally, there is a risk of discomfort or even cybersickness associated with VR usage [14] and the development of VR simulations is complex and requires trained staff [15].

While VR has become widely adopted as a training tool in medicine, its use as an assessment instrument is just emerging. It is plausible that the benefits of VR in training also apply when it is used for assessment purposes. Moreover, the high scalability of VR and its potential for objectivity, as seen in VR simulator assessments in surgery with performance metrics [16], could offer additional advantages.

Given the evident advantages of VR, integrating it into routine assessments—particularly in undergraduate nursing and medical education—appears highly promising. This study seeks to investigate the potential of VR assessments, defined here as a broad range of evaluative methods conducted within or supported by HMD VR environments. An example includes practical exams with a virtual patient, where students diagnose and manage conditions in real time. The focus was not on outcome assessments for research purposes, but rather on practical assessments intended for direct use with nursing and/or medical students. Therefore, the aim of this systematic review was to explore how HMD VR is being used as an assessment tool for nursing and/or medical students. Specifically, this review explored the settings and competencies assessed by or through VR and the taxonomic level of the assessments, as well as the practical considerations associated with implementing VR in assessments.

## Materials and methods

This preregistered systematic review (PROSPERO, CRD42023490861) is reported according to the Preferred Reporting Items for Systematic Review and Meta-Analysis (PRISMA) guideline.

### Eligibility criteria

To reflect the broad application of VR for assessment purposes (e.g., evaluating skills or knowledge) among nursing and or medical students, the following inclusion criteria were established:

All studies that conducted assessments of nursing and or medical students while they were using immersive VR, defined strictly as VR facilitated through an HMD (VR headset) were included, regardless of the original study’s defined outcomes. These assessments could involve either virtual environments or 360° video-based experiences, provided they included some form of evaluation of the students—whether conducted by a human examiner or the VR system itself.

The exclusion criteria were as follows: (1) other healthcare students, (2) other forms of VR, not facilitated by an HMD, such as screen-based, or (3) other technologies such as Mixed Reality (MR) or Augmented Reality (AR). (4) Reviews, trial protocols, conference papers or letters, and (5) all reports in languages other than English or German were also excluded.

### Search strategy

A librarian specialized in systematic literature searches was involved in initiating and reviewing the search string (See Additional File 1). The search strategy involved combining multiple terms, as well as MeSH terms, related to nursing or medical education, immersive/HMD VR, and assessment using the Boolean operator OR. These individual search strings were then conducted separately and subsequently combined using the operator AND. The systematic literature search was performed in December 2023 in Embase, PubMed, PsycINFO, Cochrane, CINAHL, and ERIC. Additionally, Google Scholar was used as a supplementary search. Manual searches were conducted by screening the reference lists of included studies as well as identified reviews that appeared eligible during the initial screening process.

The search was restricted to articles published after 2015, given the widespread availability of commercially accessible, all-in-one HMDs that operate independently without external devices since 2016 [17].

### Study selection and data extraction

The platform Rayyan (http://rayyan.qcri.org*)* was utilized to identify duplicates and efficiently search for relevant studies. To determine if a study met the review’s inclusion criteria, two independent reviewers (AN, FB) initially screened title and abstract. After the initial screening, all studies not directly excluded by both reviewers underwent full-text screening and were tested for inclusion by the same reviewers (AN, FB). Any disagreements between reviewers were resolved through discussions to reach a consensus.

The data extraction was subsequently carried out by a single reviewer (AN), followed by independent double-checking by another reviewer (FB).

### Data items

For the data extraction, information about the following topics was gathered: title, author, publication date and journal, design, country, objective, population, effective participants (only VR Intervention), inclusion and exclusion criteria, intervention, hardware and software, introduction to VR, VR assessment methods and the nature of competencies being assessed. The results were discussed among the author team.

### Quality assessment

To assess the quality of the included studies, the Medical Education Research Study Quality Instrument (MERSQI) was used, which is a useful and reliable tool to assess the quality of medical education research [18]. The MERSQI has a total of ten items over six domains: Study design, sampling, type of data, validity of evaluation instrument, data analysis and outcomes. The scale allows a maximum of 18 points. Analogous to Goldenberg et al., this work used a score of 14 or higher as an indicator of high quality [19]. The studies were independently rated by two reviewers (AN, FB), and discrepancies in every item were addressed through discussion.

### Synthesis method

Content analysis was employed to synthesize the data, utilizing a deductive approach [20]. All studies were coded for settings and competencies including tasks, taxonomic level, and practical considerations. Competencies were additionally coded according to the Accreditation Council for Graduate Medical Education (ACGME) core competencies framework including Interpersonal and Communication Skills, Patient Care, Medical Knowledge, Practice-Based Learning and Improvement, Professionalism, and Systems-based Practice [21]. The taxonomic level of assessments was coded following the well-known framework from Miller, which progresses from *knowledge* (Knows) to *competence* (Knows how), *performance* (Shows how), and finally, *action* (Does) [22]. The coding process was carried out using MAXQDA (Release 22.8.0) by a single reviewer (AN), after which the categories were discussed within the author team.

## Results

### Study selection

**Fig. 1:**
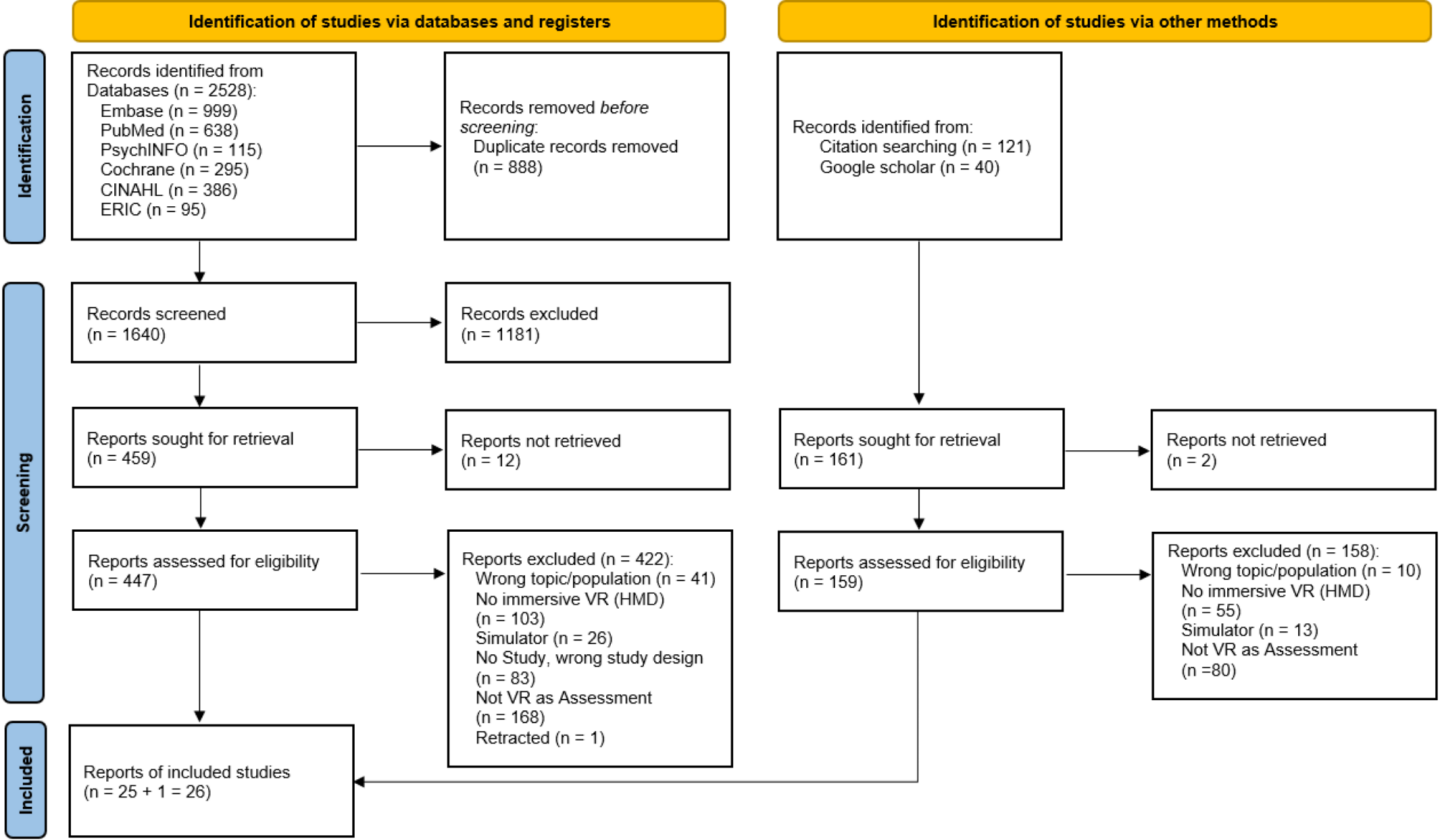
PRISMA flow diagram

A total of 2528 studies were initially found, with 888 duplicates removed (Fig. 1). Following exclusion based on title and abstract (1181 studies), 459 full-text studies were assessed, resulting in the inclusion of 25 studies. An additional search yielded 161 studies, with one meeting the inclusion criteria. In total, 26 studies were included in the final analysis (details about the fulfillment of the inclusion criteria are shown in Additional File 2).

### Study characteristics and quality

One study was conducted in 2017 [23]; while the remainder were conducted in 2020 or later (see Table 1). Studies were conducted in Europe (*n* = 9) [11, 23–30], Asia (*n* = 9) [31–39], North America (*n* = 7) [40–46], and Australia (*n* = 1) [47]. Eight studies (31%) used the VR assessment as a primary or secondary outcome [11, 28, 30, 32, 40, 42, 44, 46]. The number of participating students in the studies (only VR intervention) varied from 10 to 207 (mean 59, SD 49.9, median 38.5, IQR 26.5–73.8). The quality of the studies varied with a MERSQI score range from 7 to 15.5 (mean 12.4, SD 2.6). Eight studies (31%) had a MERSQI score over 14 indicating high quality. Please refer to the Additional File 3 for the detailed information regarding the MERSQI. We also included studies with lower quality, as our primary focus was on the VR assessment. In studies where VR was compared to other modalities, assessments of learning outcomes were often conducted in different formats, such as on mannequins [e.g., 24] or through pre/post-tests [e.g., 41]. Additionally, some studies did not focus on performance but instead evaluated factors such as workload, or usability [e.g., 30,36]. The inclusion of these studies was therefore not based on their quality but rather on their relevance to our exploration of VR as an assessment tool.

**Table 1:** Overview of included studies

Author, year Students (*n* in VR)	Setting	Competencies	Task	Taxonomic level*	Automated yes/no	MERSQI
Anbro et al., 2020 [45] Med. and nurs. (105)	Emergency setting	IC	360° Video: Serve as a recorder of the event by providing verbal checkbacks (i.e., verbal confirmation of procedural steps)	Knowledge	No	12.5
Andersen et al., 2021 [24] Med. (10)	Not specified	PC	Skill: Ultrasound-guided peripheral venous cannulation	Performance	Yes	13.5
Azher et al., 2023 [46] Nurs. (29)	Emergency setting	PC, IC	Clinical scenario: Management patient with acute anxiety	Performance	Yes	13.5
Berg & Steinsbekk, 2020 [25] Med. and nurs. (149)	Emergency setting	PC	Clinical scenario: assess patient according to ABCDE scheme (e.g. airway, breathing, circulation, disability, environment), document observations (Single-player)	Performance	Yes	15.5
Berg & Steinsbekk, 2021 [26] Med. and nurs. (146)	Emergency setting	PC	Clinical scenario: assess patient according to ABCDE scheme (e.g. airway, breathing, circulation, disability, environment, document the observations. (Multi-player)	Performance	Yes	15.5
Chao et al., 2021 [39] Nurs. (22)	Not specified	MK	360° Video; MCQ: Nasogastric tube feeding	Knowledge	Yes	15.5
Chou et al., 2023 [38] Nurs. (42)	Not specified	IC	Clinical scenario: Nurse-patient communication: self-introduction, nurse–patient relationship establishment, interaction, and medical history collection	Performance	Yes	13.5
Feeley et al., 2022 [27] Med. (28)	(Orthopedic) Surgery	PC	Procedure: Total knee replacement	Performance	Yes	13.5
Hollister et al., 2022 [44] Med. (81)	Genetics	MK, IC	Clinical scenario: Responding to patient’s medical concerns and questions (e.g. explanation of genetic test results, causes of fatigue); verbal reflections	Performance	No	15.5
Jacobs et al., 2023 [28] Med. (14)	Emergency setting	PC, MK	Clinical scenario: Sepsis identification and management	Performance	Yes	10
Knudsen et al., 2023 [30] Med. (59)	Emergency setting	MK	360° videos: 5 emergency medicine scenarios; MCQ: e.g. Procedure– what to do next	Competence	Unclear	12.5
Lau et al., 2023 [37] Nurs. (29)	Not specified	PC	Skill: subcutaneous injection and intravenous therapy	Performance	Yes	8
Lee et al., 2020 [36] Nurs. (60)	Mental health (schizophrenia)	MK, IC	360° videos: 5 schizophrenia scenarios; Quiz: adequate scenario-based reaction (e.g. identify harmful objects)	Competence	Unclear	9
Lietz et al., 2023 [11] Med. (35)	Pediatric emergency setting	PC, IC	Clinical scenario: Management of a pediatric patient with hypovolemic shock; communication with mother / assistant	Performance	Yes	14.5
Mahling et al., 2023 [29] Med. (129)	Emergency setting	PC, IC	Clinical scenario: 4 emergencies (e.g. performing examinations, lab tests, adequate communication with VP/support staff)	Performance	Yes	11
Mansoory et al., 2021 [35] Med. (50)	Emergency setting	MK	MCQ: Coma (e.g. provide initial diagnosis– Glasgow coma scale)	Knowledge	Unclear	11.5
Park & Kim, 2023 [34] Nurs. (30)	(Prehospital) Emergency setting	PC	Clinical scenario: perform first triage in a mass casualty incident (20 casualties)	Performance	Unclear	9
Perron et al., 2021 [47] Med. (23)	Pediatric emergency setting	PC, MK	Clinical scenario: Pediatric cardiopulmonary resuscitation (e.g. check for a response, performing adequate chest compressions and defibrillation)	Performance	Unclear	8
Siah et al., 2022 [33] Nurs. (207)	Perioperative setting	MK	Clinical scenario: Handling in/with sterile environment and sharp instruments (e.g. passing of sterile instruments; management of sharps injury); Quiz	Competence	Yes	9.5
Smith et al., 2021 [43] Nurs. (61)	Disaster education	MK	Skill: decontamination (e.g. donning appropriate PPE, decontaminating patient); MCQ	Knowledge	Yes	12.5
Traister, 2023 [42] Nurs. (33)	Mental health (Anxiety)	IC	Clinical scenario: Communication with anxious patient (e.g. determining the cause of patient’s acute anxiety, therapeutic communication techniques)	Performance	Yes	13
Wan et al., 2024 [32] Med. (20)	(Maxillofacial) Surgery	PC	Procedure: Bimaxillary orthognathic surgery	Performance	Yes	14.5
Wilson et al., 2017 [23] Med. (15)	Ophthalmology	PC, MK	Skill: Performing retinal examination; MCQ: identify abnormalities in images	Performance	Yes	7
Wu et al., 2022 [31] Nurs. (53)	Pediatric emergency setting	PC, MK, IC	Clinical scenario: Managing pediatric seizure (e.g. check vital sign, provide family education); MCQ: select correct seizure type	Performance	Yes	14.5
Zackoff et al., 2020 [40] Med. (78)	Pediatric emergency setting	PC, MK, IC	Clinical scenario: Managing pediatric bronchiolitis in three simulations (1) no distress, (2) respiratory distress, (3) impending respiratory failure	Performance	No	14.5
Zackoff et al., 2021 [41] Med. (26)	Pediatric emergency setting	PC, MK, IC	Clinical scenario: Managing pediatric viral bronchiolitis (e.g. clinical assessment and examination, therapeutic steps)	Performance	No	12.5

Note. Med.: medical; Nurs.: nursing; VR: Virtual Reality; MERSQI: Medical Education Research Study Quality Instrument; IC: Interpersonal and Communication Skills; PC: Patient Care; MK: Medical Knowledge; ABCDE: Airway, Breathing, Circulation, Disability, Exposure; MCQ: multiple choice questions; VP: virtual patient; PPE: personal protective equipment

*According to Miller [22]

### Settings and competencies

**Fig. 2:**
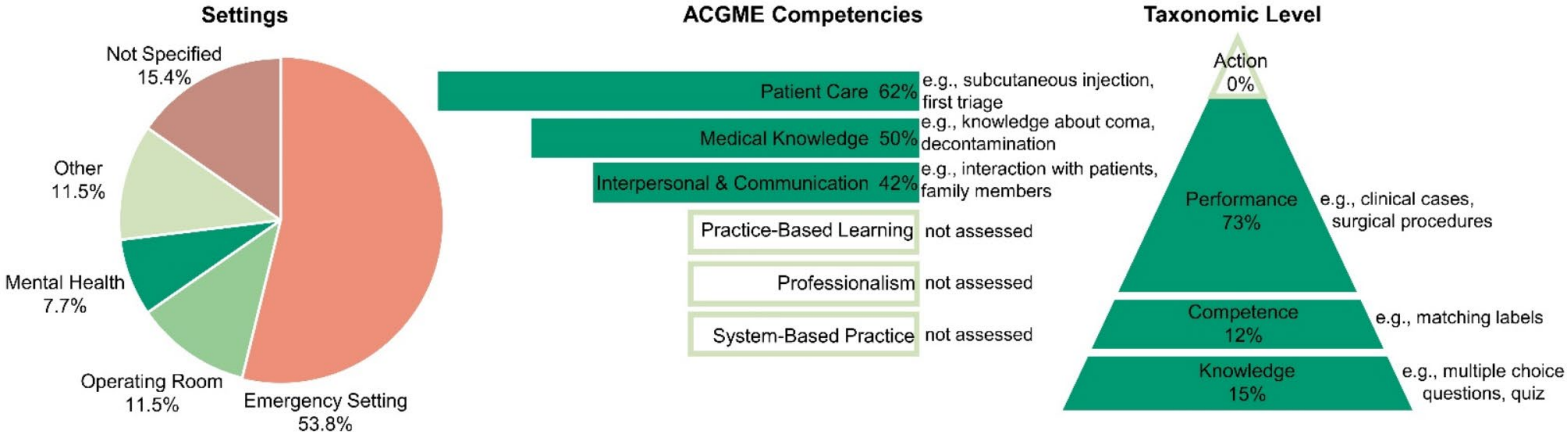
Summary of results: Settings, competencies and taxonomic levels. On the left, a pie chart illustrates the distribution of study settings. In the center, the Accreditation Council for Graduate Medical Education (ACGME) competencies are presented, with the size of the bars indicating the frequency with which each competency was assessed. On the right, a pyramid diagram displays the taxonomic levels, with the shape and size reflecting the frequency of their application. Additionally, examples of tasks associated with each competency are provided, as well as examples of how the different taxonomic levels were applied in the studies

Fourteen studies (54%) examined emergency settings [25, 26, 28–30, 35, 45, 46], including five (19%) that focused on pediatric emergencies [11, 31, 40, 41, 47] and one on prehospital emergencies with mass casualty incidents [34]. In addition, three studies (12%) were conducted in the operating room, two of which specifically addressed surgery [27, 32], while one covered general perioperative practice such as sterile procedures [33]. Furthermore, two assessments (8%) were related to mental health [36, 42], one explored genetics [44], one on disaster education [43], and one on ophthalmology [23]. The four remaining studies (15%) did not specify a particular setting (see Table 1 or Fig. 2) [24, 37–39].

The VR assessments targeted three of the six ACGME core competencies: Patient Care (62%), Interpersonal and Communication Skills (50%), and Medical Knowledge (42%). For an overview, see Fig. 2. Patient care was assessed to varying degrees, ranging from individual skills such as peripheral venous catheter placement [24], performing retinal examination [23], subcutaneous injection, and intravenous therapy [37] or first triage [34] to entire procedures such as history taking and clinical assessment as well as treatments (e.g., pediatric seizure or respiratory management) [11, 25, 26, 28, 29, 31, 40, 41, 46] and cardiopulmonary resuscitation [47], or entire surgical procedures [27, 32]. Interpersonal and communication skills were examined, through the evaluation of interactions with (anxious) patients [38, 42, 44], family members [11, 31], or inter-professional communication [11, 29, 36, 40, 41, 45, 46]. Medical knowledge assessments covered a variety of topics, including knowledge about coma [35], nasogastric tube feeding [39], decontamination [43], ophthalmology [23] and others [28, 30, 31, 33, 36, 40, 41, 44, 47], as detailed in Table 1. Some studies also assessed multiple competencies simultaneously [11, 23, 28, 29, 31, 36, 40, 41, 44, 46, 47]. Notably, competencies such as practice-based learning and improvement, professionalism, and systems-based practice were either not assessed or not explicitly addressed in these studies.

### Taxonomic level of assessment

The taxonomic level of the VR assessment according to Miller’s pyramid is shown in Table 1; Fig. 2. Three of the four studies that tested *knowledge* (15%) used multiple-choice questions (MCQs) or quizzes [35, 39, 43]. The questions were presented to the participants in VR and had to be answered by using the hand-held controllers. Anbro et al. used verbal checkbacks instead [45]. In the three studies (12%) that assessed *competence*, also multiple-choice questions (MCQs) were used [30, 33, 36]. However, additional methods such as matching quizzes, like matching the correct labels, as well as tasks like “rearrangement of sequences” and scenario-based assessments, were also employed to assess not only knowledge but also to evaluate whether the knowledge was understood and could be applied in this specific context. The remaining 19 *performanc*e level studies (73%) used clinical cases such as Objective Structured Clinical Examinations (OSCE) or surgical procedures [11, 23–29, 31, 32, 34, 37, 38, 40–42, 44, 46, 47]. The *action* level, was absent, as it would ideally occur during clinical practice [22].

All VR assessments were assumed to be formative (low stakes), even though seven studies (27%) integrated VR interventions into courses or curricula [25, 26, 29, 31, 40, 45, 46]. However, participation in these studies was voluntary, suggesting formative assessment. A detailed overview of the key findings from the chapters on settings and competencies, and taxonomic levels, can be found in Fig. 2.

### Practical considerations

Two different ways were used to implement VR: in 17 studies (65%) as an automated and independent assessment tool, meaning it performed the evaluation without human intervention [11, 23–29, 31–33, 37–39, 42, 43, 46], and four times (15%) as a supporting assessment tool [40, 41, 44, 45], where the assessment took place in VR but the evaluation was carried out by human examiners instead (see Table 1). This could not be determined in the other studies, as it was not clearly described whether the evaluation was conducted by the VR system itself or by an external examiner. An example of a VR automated assessment is described by Traister, the software used a dashboard that provides feedback ranging from 0 to 100%, showing the individual’s average score, percentages related to skill performance across technical skills, communication, teamwork, and timing [42]. The calculation method for the feedback was not always explicitly described. When VR was used as a supporting tool, the VR scenarios were evaluated by an external evaluator either directly or via recording and transcription using a checklist.

Regarding hardware, five studies (19%) did not specify the HMDs used [27, 33–35, 42], 14 studies (54%) utilized Oculus (Meta, California, United States of America) [11, 25, 26, 28–31, 36, 37, 40, 41, 43, 46, 47] and six (23%) used HTC devices (HTC Corporation, Taoyuan City, Taiwan) [24, 32, 38, 39, 44, 45]. In the oldest study two HMDs were used, both requiring a smartphone for functionality [23]. Subsequent studies utilized all-in-one HMDs, eliminating the need for smartphones or laptops. The necessity of an external computer with all-in-one HMDs varied based on the software. Three studies (12%) mentioned the use of gaming laptops [24, 29, 47], while others did not report or utilize them. For additional analyses, one study incorporated eye trackers [45].

The software used in most studies was custom-made, while six (23%) utilized commercially available options: VR USGIVA 1.0 (VitaSim, Odense, Denmark) [24], Gogglemind Ltd (Cardiff, UK) [30], Precision OS virtual reality platform (Vancouver, Canada) [27] and three used the Oxford Medical Simulation Ltd. (London, UK) [29, 42, 46]. It is assumed that studies not specifying the software used custom-developed software, as commercial software would likely be mentioned. However, this remains uncertain. Four studies (15%) utilized 360-degree videos, integrating them with interactive elements like multiple-choice questions [30, 36, 39, 45]. See Additional File 4 for more information about hardware and software.

In studies focusing on clinical scenarios, virtual patient (VP) avatars were utilized. Berg and Steinsbekk described the VP’s visual responses such as eye blinking, head movement, and mouth opening and closing [26]. Additionally, dynamic clinical parameters such as blood pressure, temperature, and oxygen saturation were simulated. However, these avatars did not provide vocal responses. The VP’s in other studies were capable of communication; Interaction with these patients was facilitated, for instance, through clicking on voice commands [28, 37], prerecorded answers prompted by the research assistant [44], or Microsoft Azure (Washington, United States) voice recognition [31]. Two studies (8%) explicitly mentioned implementing haptic feedback through vibration in hand controllers, allowing users to feel the pulse on the wrist and respiratory intake, when placing the hand on the chest [25, 26]. This feedback might have been present in other studies but was not explicitly described.

All studies except for three (12%) mentioned a form of VR-tutorial or orientation in VR [35, 40, 47]. The introductions to the VR experiences showed a lot of variation, from verbal instructions, slideshows, pre-recorded videos or tutorials directly in VR. One study further described that they explained cybersickness and safety management purposes [34].

Technical challenges were scarcely reported, but several studies noted that an assistant supervised the VR simulation to intervene in case of potential technical issues. Some studies have also investigated potential side effects of VR. Three studies (12%) indicated no to minimal side effects such as cybersickness [31, 38, 39], while two (8%) mentioned that cybersickness and physical discomfort occurred, but did not specify their severity [29, 37]. The side effect reported the most appeared to be dizziness. Additionally, specific exclusion criteria were mentioned (e.g. epilepsy, vestibular disorders, pregnancy, or adverse effects such as nausea after using VR equipment) [29, 31, 33, 38, 39, 44].

## Discussion

This systematic review outlines a variety of VR assessments used in nursing and medical undergraduate education across different settings, mainly in situation that are difficult to replicate such as (pediatric) emergencies. The studies focused on core competencies, including Patient Care, Interpersonal and Communication Skills, and Medical Knowledge. Taxonomic levels ranged from *knowledge* (Knows) to *performance* (Shows how). Additionally, the review sheds light on the practical considerations surrounding VR implementation, including hardware and software variations.

Assessments using VR were primarily employed in situations that are difficult to replicate in real life. This phenomenon is also observed in VR training, highlighting a significant advantage of VR technology [10]. Competencies such as Practice-based Learning and Improvement, Professionalism, and Systems-based Practice were either not assessed or not explicitly addressed in the included studies. Assessment should cover all aspects of medical competence. It is acknowledged that competencies such as Systems-based Practice are less effectively captured using conventional methods too [21]. One study implied, but did not explicitly assess, Professionalism and System-based Practice related to different races and genetic test reports [44]. Similarly, other studies trained empathy, which could also be considered part of Professionalism, but did not explicitly test it through VR [48]. Therefore, it is worth exploring the extent to which VR can address this issue.

Apart from *action*, all other taxonomic levels can be tested in VR. Although one may argue that VR can simulate high-fidelity clinical scenarios that approximate *action* to some extent, it would need to assess performance in real practice rather than in a simulated environment to fully align with Miller’s concept of *action*, which refers specifically to work-based assessments [22, 49]. It is notable that some studies conducted VR assessments below *performance* level, although the obvious potential lies there. This may be due to initial testing purposes, with less attention paid to the pedagogical implications. For further use it should be considered whether the effort required to implement MCQs in VR, which can be tested in written form, justifies their use in VR [50, 51]. Additionally, it is important to determine which learning objectives should be assessed in VR and train accordingly to achieve alignment between learning objectives, teaching, and assessment [52, 53].

Although some studies have described the integration of VR into the curriculum (e.g [29]), assessments are likely to have remained formative due to the study setting, primarily serving training purposes. Unfortunately, little was discussed regarding feasibility, as implementing VR for an entire cohort differs significantly from just a limited number of study participants. However, one study recently integrated VR into an exam for one cohort and demonstrated its feasibility, both technically and organizationally, suggesting that such an implementation could be possible [54].

One of the advantages of VR is the automated assessment, where the system directly evaluates performance, a concept already applied in the field of surgery [16]. In the context of undergraduate studies, automated assessment could be employed in the OSCE, addressing the significant personal demands on clinicians and the intense mental effort required of examiners. The implementation of automated VR assessment could also facilitate the attainment of objectivity [55, 56]. Tasks, such as accurately auscultating the lungs or appropriately selecting laboratory results, could be automatically evaluated and marked off by the VR software based on a predefined OSCE checklist. This would enable examiners to allocate their cognitive resources more effectively, allowing them to focus on higher-order skills, such as communication. The objectivity that automated VR assessments could offer may also benefit students by providing consistent feedback that is not subject to examiner variability. However, it is important to note that students might be skeptical about the reliability of such assessments [57].

In recent years, HMDs have undergone significant advancements and are now more affordable to purchase. However, commercially available software may be costly and lack customization. Creating custom software can also be time-consuming, resource-intensive, and expensive [58, 59]. Additionally, integrating sophisticated VR software alone does not guarantee successful assessment; validity must be carefully considered. The potential of VR assessment remains underutilized when, for example, complex VR scenarios are designed where learners engage in detailed action sequences, only to be assessed at the end by answering a single multiple-choice question with a controller click. Simply integrating new technology into a training program does not inherently enhance its value; instead, it is crucial to ensure that the technology is thoughtfully integrated into a comprehensive pedagogical strategy. Close collaboration between clinicians, medical educators and software engineers during development is critical to ensure effective assessment tools [6].

Technical issues were rarely reported, but having personnel available to address them seems crucial [60]. Nearly all studies mentioned introducing students to VR, which is essential, especially for assessments, to ensure performance issues are not attributed to unfamiliarity with the system. Additionally, consideration should be given to exclusion criteria, such as how to accommodate such students especially in summative assessments [61].

### Strengths and weaknesses of this review

The role of VR, especially as an assessment tool, is an emerging field covered in this review. The search strategy was developed and validated by a specialized librarian, and the review was conducted with methodological rigor. Additionally, it has an interprofessional author team that includes experts from medical education. Categorizing competencies and assessment methods was occasionally challenging due to vague descriptions in some studies, likely because assessment was not their primary focus. To ensure a comprehensive overview, we included studies regardless of whether VR assessments were used as outcome measures. This approach was chosen to avoid limiting the scope to a small number of studies and to better align with our aim of capturing the diverse ways in which VR can be utilized. By doing so, we aimed to highlight the broad potential of VR beyond its role as a tool for measuring outcomes.

### Conclusion and future directions

This review provides insight into using VR for assessment, especially in situations that are difficult to replicate like emergencies. The primary focus of VR assessments is on core competencies such as Patient Care, Interpersonal Communication, and Medical Knowledge, with a focus on performance-level tasks. While VR shows promise in automating assessments and reducing examiner cognitive load, there are many technical details such as software, costs and feasibility to consider. To maximize its effectiveness as an educational and evaluative tool, it is crucial that VR assessments align with learning objectives and teaching methods, guided by the principles of constructive alignment. Automated assessments also hold significant promise from a practical perspective. A challenge in practical exams, such as OSCEs, is the burden placed on examiners. Future research should explore the potential of VR to enable objective, automated assessments. Additionally, it is essential to investigate how VR assessments can be seamlessly integrated into existing curricula. Research should also examine the feasibility and cost-effectiveness of scaling VR assessments for larger cohorts. Finally, exploring the integration of VR with advanced technologies, such as Artificial Intelligence, could open new possibilities for enhancing automation and personalizing assessments to individual learners.

## Electronic supplementary material

Below is the link to the electronic supplementary material.

Supplementary Material 1: Additional File 1: Search Terms for each database.

Supplementary Material 2: Additional File 2: Comprehensive justifications for study inclusion in the systematic review.

Supplementary Material 3: Additional File 3: MERSQI Scores for each study.

Supplementary Material 4: Additional File 4: Overview of used hardware and software.

## Data Availability

No datasets were generated or analysed during the current study.

## Declarations

This systematic review was registered in PROSPERO (CRD42023490861) and utilized the MERSQI Tool instead of the Cochrane Risk-of-Bias-Tool (as initially registered) for assessing data quality due to the inclusion of a wide range of study designs.

## Abbreviations

ABCDE: Airway, Breathing, Circulation, Disability, Exposure
ACGME: Accreditation Council for Graduate Medical Education
HMD: Head-mounted Display
IC: Interpersonal and Communication Skills
MCQ: Multiple Choice Questions
Med: Medical
MK: Medical Knowledge
Nurs: Nursing
OSCE: Objective Structured Clinical Examination
PC: Patient Care
PPE: Personal Protective Equipment
PRISMA: Preferred Reporting Items for Systematic Review and Meta-Analysis
VP: Virtual Patient
VR: Virtual Reality
