# Supplementary material for: Virtual reality for assessment in undergraduate nursing and medical education – a systematic review
Source: BMC Med Educ. 2025 Feb 22;25:292. doi: 10.1186/s12909-025-06867-8 (PMC11846274; doi:10.1186/s12909-025-06867-8)
Supplement: Supplementary file 1 — Supplementary Material 1: Additional File 1: Search Terms for each database. [file 12909_2025_6867_MOESM1_ESM.docx]

**Additional File 1: Search Terms for each database**

**Table of content**

[Embase 2](#_Toc163488366)

[PubMed 3](#_Toc163488367)

[PsycInfo 4](#_Toc163488368)

[Cochrane 5](#_Toc163488369)

[CINAHL 6](#_Toc163488370)

[Eric 7](#_Toc163488371)

## Embase

| 06.12.2023 | | |
| --- | --- | --- |
| **Component** | **Terms** | **Results** |
| Population (nursing and medical students) | 1 or 2 | 412’525 |
|  | 1 = medical education/ or nursing education/  2 = ("medical education" or "medical student*" or "medical curricul*" or "nursing education" or "nursing student*" or "nursing curricul*").ti,ab,id. |  |
| Intervention (immersive Virtual Reality) | 4 or 5 | 46’681 |
|  | 4 = virtual reality/ or simulation-based assessment/ or virtual environment/  5 =("virtual realit*” or HMD or "head mounted display*" or "virtual environment*" or "immersive virtual reality” or “immersive vr” or vr).ti,ab,id. |  |
| Outcome (Performance assessment) | 7 or 8 | 9’408’264 |
|  | 7 = educational measurement/  8= (performance or assessment* or osce or exam* or evaluation or appraisal or "objective measure*").ti,ab,id. |  |
| Full search strategy | 3 and 6 and 9 | 1820 |
| Filter | 2016 - now | 999 |

## PubMed

| 05.12.2023 | | |
| --- | --- | --- |
| **Component** | **Terms** | **Results** |
| Population (nursing and medical students) | ((("Education, Medical"[Mesh:NoExp]) OR "Education, Medical, Undergraduate"[Mesh]) OR "Education, Nursing"[Mesh]) OR ("medical education"[Title/Abstract] OR "medical student*"[Title/Abstract] OR "medical curricul*"[Title/Abstract] OR "nursing education"[Title/Abstract] OR "nursing student*"[Title/Abstract] OR "nursing curricul*"[Title/Abstract]) | 258’778 |
| Intervention (immersive Virtual Reality) | ("Virtual Reality"[Mesh]) OR ("virtual realit*"[Title/Abstract] OR HMD[Title/Abstract] OR "head mounted display*"[Title/Abstract] OR "virtual environment*"[Title/Abstract] OR "immersive virtual reality"[Title/Abstract] OR "immersive vr"[Title/Abstract] OR vr[Title/Abstract]) | 29’538 |
| Outcome (Performance assessment) | (("Educational Measurement"[Mesh:NoExp]) OR "Academic Performance"[Mesh]) OR performance[Title/Abstract] OR assessment*[Title/Abstract] OR osce[Title/Abstract] OR exam*[Title/Abstract] OR evaluation[Title/Abstract] OR appraisal[Title/Abstract] OR "objective measure*"[Title/Abstract] | 7’108’121 |
| Full search strategy | ((((("Education, Medical"[Mesh:NoExp]) OR "Education, Medical, Undergraduate"[Mesh]) OR "Education, Nursing"[Mesh]) OR ("medical education"[Title/Abstract] OR "medical student*"[Title/Abstract] OR "medical curricul*"[Title/Abstract] OR "nursing education"[Title/Abstract] OR "nursing student*"[Title/Abstract] OR "nursing curricul*"[Title/Abstract])) AND (("Virtual Reality"[Mesh]) OR ("virtual realit*"[Title/Abstract] OR HMD[Title/Abstract] OR "head mounted display*"[Title/Abstract] OR "virtual environment*"[Title/Abstract] OR "immersive virtual reality"[Title/Abstract] OR "immersive vr"[Title/Abstract] OR vr[Title/Abstract]))) AND ((("Educational Measurement"[Mesh:NoExp]) OR "Academic Performance"[Mesh]) OR performance[Title/Abstract] OR assessment*[Title/Abstract] OR osce[Title/Abstract] OR exam*[Title/Abstract] OR evaluation[Title/Abstract] OR appraisal[Title/Abstract] OR "objective measure*"[Title/Abstract]) | 986 |
| Filter | 2016 - now | 638 |

## PsycInfo

| 05.12.2023 | | |
| --- | --- | --- |
| **Component** | **Terms** | **Results** |
| Population (nursing and medical students) | 1 or 2 | 45’584 |
|  | 1 = medical education/ or nursing education/  2 = ("medical education" or "medical student*" or "medical curricul*" or "nursing education" or "nursing student*" or "nursing curricul*").ti,ab,id. |  |
| Intervention (immersive Virtual Reality) | 4 or 5 | 17’462 |
|  | 4 = virtual reality/ or simulation-based assessment/ or virtual environment/  5 =("virtual realit*” or HMD or "head mounted display*" or "virtual environment*" or "immersive virtual reality” or “immersive vr” or vr).ti,ab,id. |  |
| Outcome (Performance assessment) | 7 or 8 | 2’131’113 |
|  | 7 = educational measurement/  8= (performance or assessment* or osce or exam* or evaluation or appraisal or "objective measure*").ti,ab,id. |  |
| Full search strategy | 3 and 6 and 9 | 192 |
| Filter | 2016 - now | 115 |

## Cochrane

| 05.12.2023 | | |
| --- | --- | --- |
| **Component** | **Terms** | **Results** |
| Population (nursing and medical students) | #1 OR #2 OR #3 OR #4 | 10’424 |
|  | 1 MeSH descriptor: [Education, Medical] this term only  2 MeSH descriptor: [Education, Medical, Undergraduate] this term only  3 MeSH descriptor: [Education, Nursing] this term only  4 (("medical education") or (medical NEXT student*) or (medical NEXT curricul*) or (nursing NEXT student*) or ("nursing education") or (nursing NEXT student*) or (nursing NEXT curricul*)):ti,ab,kw |  |
| Intervention (immersive Virtual Reality) | #6 OR #7 | 7’806 |
|  | 6 MeSH descriptor: [Virtual Reality] this term only  7 ((HMD) or ("head mounted display") or (virtual NEXT environment*) or (virtual NEXT realit*) or (“immersive vr”) or (vr) or ("head mounted displays")):ti,ab,kw |  |
| Outcome (Performance assessment) | #9 OR #10 OR #11 | 1’120’867 |
|  | 9 MeSH descriptor: [Educational Measurement] this term only  10 MeSH descriptor: [Academic Performance] this term only  11 ((performance) or (assessment*) or (osce) or (exam*) or (evaluation) or (appraisal) or (objective NEXT measure*)):ti,ab,kw |  |
| Full search strategy | #5 AND #8 AND #12 | 425 |
| Filter | 2016 - now | 295 |

## CINAHL

| 05.12.2023 | | |
| --- | --- | --- |
| **Component** | **Terms** | **Results** |
| Population (nursing and medical students) | S1 OR S2 OR S3 | 131’439 |
|  | S1 (MH "Education, Medical")  S2 (MH "Education, Nursing")  S3 TI ( "medical education" or "medical student*" or "medical curricul*" or "nursing education" or "nursing student*" or "nursing curricul*" ) OR AB ( "medical education" or "medical student*" or "medical curricul*" or "nursing education" or "nursing student*" or "nursing curricul*" ) |  |
| Intervention (immersive Virtual Reality) | S5 OR S6 | 13’991 |
|  | S5 (MH "Virtual Reality")  S6 TI ( "virtual realit*” or HMD or "head mounted display*" or "virtual environment*" or "immersive virtual reality” or “immersive vr” or vr ) OR AB ( "virtual realit*” or HMD or "head mounted display*" or "virtual environment*" or "immersive virtual reality” or “immersive vr” or vr ) |  |
| Outcome (Performance assessment) | S8 OR S9 OR S10 | 1’605’911 |
|  | S8 (MH "Educational Measurement")  S9 (MH "Academic Performance")  S10 TI (performance or assessment* or osce or exam* or evaluation or appraisal or "objective measure*" ) OR AB (performance or assessment* or osce or exam* or evaluation or appraisal or "objective measure*" ) |  |
| Full search strategy | S4 AND S7 AND S11 | 563 |
|  | ( (MH "Education, Medical") OR (MH "Education, Nursing") OR ( TI ( "medical education" or "medical student*" or "medical curricul*" or "nursing education" or "nursing student*" or "nursing curricul*" ) OR AB ( "medical education" or "medical student*" or "medical curricul*" or "nursing education" or "nursing student*" or "nursing curricul*" ) ) ) AND ( (MH "Virtual Reality") OR ( TI ( "virtual realit*” or HMD or "head mounted display*" or "virtual environment*" or "immersive virtual reality” or “immersive vr” or vr ) OR AB ( "virtual realit*” or HMD or "head mounted display*" or "virtual environment*" or "immersive virtual reality” or “immersive vr” or vr ) ) ) AND ( (MH "Educational Measurement") OR (MH "Academic Performance") OR ( TI (performance or assessment* or osce or exam* or evaluation or appraisal or "objective measure*" ) OR AB (performance or assessment* or osce or exam* or evaluation or appraisal or "objective measure*" ) ) ) |  |
| Filter | 2016 - now | 386 |

## Eric

| 05.12.2023 | | |
| --- | --- | --- |
| **Component** | **Terms** | **Results** |
| Population (nursing and medical students) | S1 OR S2 | 18’103 |
|  | S1 DE "Medical Education" OR DE "Nursing Education"  S2 TI ( "medical education" or "medical student*" or "medical curricul*" or "nursing education" or "nursing student*" or "nursing curricul*" ) OR AB ( "medical education" or "medical student*" or "medical curricul*" or "nursing education" or "nursing student*" or "nursing curricul*" ) |  |
| Intervention (immersive Virtual Reality) | S4 OR S5 | 11’298 |
|  | S4 DE "Computer Simulation"  S5 TI ( "virtual realit*” or HMD or "head mounted display*" or "virtual environment*" or "immersive virtual reality” or “immersive vr” or vr ) OR AB ( "virtual realit*” or HMD or "head mounted display*" or "virtual environment*" or "immersive virtual reality” or “immersive vr” or vr ) |  |
| Outcome (Performance assessment) | S7 OR S8 | 781’279 |
|  | S7 DE "Academic Achievement"  S8 TI (performance or assessment* or osce or exam* or evaluation or appraisal or "objective measure*" ) OR AB (performance or assessment* or osce or exam* or evaluation or appraisal or "objective measure*" ) |  |
| Full search strategy | S4 AND S7 AND S11 | 186 |
|  | ( DE "Medical Education" OR DE "Nursing Education" OR ( TI ( "medical education" or "medical student*" or "medical curricul*" or "nursing education" or "nursing student*" or "nursing curricul*" ) OR AB ( "medical education" or "medical student*" or "medical curricul*" or "nursing education" or "nursing student*" or "nursing curricul*" ) ) ) AND ( DE "Computer Simulation" OR ( TI ( "virtual realit*” or HMD or "head mounted display*" or "virtual environment*" or "immersive virtual reality” or “immersive vr” or vr ) OR AB ( "virtual realit*” or HMD or "head mounted display*" or "virtual environment*" or "immersive virtual reality” or “immersive vr” or vr ) ) ) AND ( DE "Academic Achievement" OR ( TI (performance or assessment* or osce or exam* or evaluation or appraisal or "objective measure*" ) OR AB (performance or assessment* or osce or exam* or evaluation or appraisal or "objective measure*" ) ) ) |  |
| Filter | 2016 - now | 95 |
