# Supplementary material for: Virtual reality for assessment in undergraduate nursing and medical education – a systematic review
Source: BMC Med Educ. 2025 Feb 22;25:292. doi: 10.1186/s12909-025-06867-8 (PMC11846274; doi:10.1186/s12909-025-06867-8)
Supplement: Supplementary file 2 — Supplementary Material 2: Additional File 2: Comprehensive justifications for study inclusion in the systematic review. [file 12909_2025_6867_MOESM2_ESM.docx]

**Additional File 3: MERSQI Scores for each study**

|  | **Study Design** | **Sampling** | | **Type of Data** | | | | **Data analysis** |  | **Outcomes** |  |
| --- | --- | --- | --- | --- | --- | --- | --- | --- | --- | --- | --- |
| **Author, year** | 1.  Study Design | 2. No of institutions studied | 3.  Response rate | 4.  Type of data | 5.  Internal structure | 6.  Content | 7. Relationships other variables | 8. Appropriateness of analysis | 9.  Complexity of analysis | 10.  Outcomes | **MERSQI Score** |
| Anbro et al., 2020[1] | 1.5 | 1 | 1.5 | 3 | 0 | 1 | 0 | 1 | 2 | 1.5 | 12.5 |
| Andersen et al., 2021[2] | 3 | 0.5 | 1.5 | 3 | 0 | 1 | 0 | 1 | 2 | 1.5 | 13.5 |
| Azher et al., 2023[3] | 2 | 0.5 | 1.5 | 3 | 0 | 1 | 1 | 1 | 2 | 1.5 | 13.5 |
| Berg & Steinsbekk, 2020[4] | 3 | 0.5 | 1.5 | 3 | 1 | 1 | 1 | 1 | 2 | 1.5 | 15.5 |
| Berg & Steinsbekk, 2021[5] | 3 | 0.5 | 1.5 | 3 | 1 | 1 | 1 | 1 | 2 | 1.5 | 15.5 |
| Chao et al., 2021[6] | 3 | 0.5 | 1.5 | 3 | 1 | 1 | 1 | 1 | 2 | 1.5 | 15.5 |
| Chou et al., 2023[7] | 3 | 0.5 | 1.5 | 1 | 1 | 1 | 1 | 1 | 2 | 1.5 | 13.5 |
| Feeley et al., 2022[8] | 3 | 0.5 | 0.5 | 3 | 0 | 1 | 1 | 1 | 2 | 1.5 | 13.5 |
| Hollister et al., 2022[9] | 3 | 0.5 | 1.5 | 3 | 1 | 1 | 1 | 1 | 2 | 1.5 | 15.5 |
| Jacobs et al., 2023[10] | 1 | 0.5 | 1.5 | 1 | 0 | 1 | 1 | 1 | 2 | 1 | 10 |
| Knudsen et al., 2023[11] | 1 | 0.5 | 1.5 | 3 | 1 | 0 | 1 | 1 | 2 | 1.5 | 12.5 |
| Lau et al., 2023[12] | 1 | 0.5 | 0.5 | 1 | 1 | 1 | 0 | 1 | 1 | 1 | 8 |
| Lee et al., 2020[13] | 1 | 0.5 | 1.5 | 1 | 1 | 1 | 0 | 1 | 1 | 1 | 9 |
| Lietz et al., 2023[14] | 3 | 0.5 | 1.5 | 3 | 0 | 1 | 1 | 1 | 2 | 1.5 | 14.5 |
| Mahling et al., 2023[15] | 1 | 0.5 | 1 | 3 | 0 | 0 | 1 | 1 | 2 | 1.5 | 11 |
| Mansoory et al., 2021[16] | 2 | 0.5 | 1.5 | 1 | 1 | 0 | 1 | 1 | 2 | 1.5 | 11.5 |
| Park & Kim, 2023[17] | 1 | 0.5 | 1.5 | 1 | 1 | 1 | 0 | 1 | 1 | 1 | 9 |
| Perron et al., 2021[18] | 1 | 0.5 | 1.5 | 1 | 0 | 1 | 0 | 1 | 1 | 1 | 8 |
| Siah et al., 2022[19] | 1 | 0.5 | 1.5 | 1 | 0 | 0 | 1 | 1 | 2 | 1.5 | 9.5 |
| Smith et al., 2021[20] | 2 | 0.5 | 1.5 | 3 | 0 | 0 | 1 | 1 | 2 | 1.5 | 12.5 |
| Traister, 2023[21] | 1.5 | 0.5 | 1.5 | 3 | 0 | 1 | 1 | 1 | 2 | 1.5 | 13 |
| Wan et al., 2024 [22] | 3 | 0.5 | 1.5 | 3 | 0 | 1 | 1 | 1 | 2 | 1.5 | 14.5 |
| Wilson et al., 2017[23] | 1 | 0.5 | 1.5 | 1 | 0 | 1 | 0 | 0 | 1 | 1 | 7 |
| Wu et al., 2022[24] | 2 | 0.5 | 1.5 | 3 | 1 | 1 | 1 | 1 | 2 | 1.5 | 14.5 |
| Zackoff et al., 2020[25] | 3 | 0.5 | 1.5 | 3 | 0 | 1 | 1 | 1 | 2 | 1.5 | 14.5 |
| Zackoff et al., 2021[26] | 1 | 0.5 | 1.5 | 3 | 1 | 0 | 1 | 1 | 2 | 1.5 | 12.5 |

1. Anbro SJ, Szarko AJ, Houmanfar RA, Maraccini AM, Crosswell LH, Harris FC, et al. Using virtual simulations to assess situational awareness and communication in medical and nursing education: A technical feasibility study. J Organ Behav Manag. 2020;(1–2):129–39.

2. Andersen NL, Jensen RO, Posth S, Laursen CB, Jørgensen R, Graumann O. Teaching ultrasound-guided peripheral venous catheter placement through immersive virtual reality: An explorative pilot study. Medicine (Baltimore). 2021;100(27):1–7.

3. Azher S, Cervantes A, Marchionni C, Grewal K, Marchand H, Harley JM. Virtual simulation in nursing education: Headset virtual reality and screen-based virtual simulation offer a comparable experience. Clin Simul Nurs. 2023;79:61–74.

4. Berg H, Steinsbekk A. Is individual practice in an immersive and interactive virtual reality application non-inferior to practicing with traditional equipment in learning systematic clinical observation? A randomized controlled trial. BMC Med Educ. 2020;20(1):123.

5. Berg H, Steinsbekk A. The effect of self-practicing systematic clinical observations in a multiplayer, immersive, interactive virtual reality application versus physical equipment: a randomized controlled trial. Adv Health Sci Educ. 2021;26(2):667–82.

6. Chao YC, Hu SH, Chiu HY, Huang PH, Tsai HT, Chuang YH. The effects of an immersive 3d interactive video program on improving student nurses’ nursing skill competence: A randomized controlled trial study. Nurse Educ Today. 2021;103:104979.

7. Chou CH, Tai HC, Chen SL. The effects of introducing virtual reality communication simulation in students’ learning in a fundamentals of nursing practicum: A pragmatic randomized control trials. Nurse Educ Pract. 2023;74:103837.

8. Feeley A, Feeley I, Lee M, Merghani K, Sheehan E. The specialty mentor effect in enhancing surgical experience of medical students: A randomised control trial. Surg Elsevier Sci. 2022;20(6):383–8.

9. Hollister B, Schopp E, Telaak S, Buscetta A, Dolwick A, Fortney C, et al. Educational considerations based on medical student use of polygenic risk information and apparent race in a simulated consultation. Genet Med. 2022;24(11):2389‐2398.

10. Jacobs C, Vaidya K, Medwell L, Old T, Joiner R. Case study of virtual reality sepsis management- instructional design and ITEM outcomes. J Vis Commun Med. 2023;1–10.

11. Knudsen MH, Breindahl N, Dalsgaard TS, Isbye D, Mølbak AG, Tiwald G, et al. Using virtual reality head-mounted displays to assess skills in emergency medicine: Validity study. J Med Internet Res. 2023;25.

12. Lau ST, Siah RCJ, Dzakirin Bin Rusli K, Loh WL, Yap JYG, Ang E, et al. Design and evaluation of using head-mounted virtual reality for learning clinical procedures: Mixed methods study. JMIR Serious Games. 2023;11.

13. Lee Y, Kim SK, Eom MR. Usability of mental illness simulation involving scenarios with patients with schizophrenia via immersive virtual reality: A mixed methods study. PLOS ONE. 2020;15(9).

14. Lietz A, Kraller J, Hoffelner A, Ritschl V, Berger A, Wagner M. Dose–response of virtual reality training of paediatric emergencies in a randomised simulation‐based setting. Acta Paediatr. 2023;112(9).

15. Mahling M, Wunderlich R, Steiner D, Gorgati E, Festl-Wietek T, Herrmann-Werner A. Virtual reality for emergency medicine training in medical school: Prospective, large-cohort implementation study. J Med Internet Res. 2023;25.

16. Mansoory M, Khazaei M, Azizi S, Niromand E. Comparison of the effectiveness of lecture instruction and virtual reality-based serious gaming instruction on the medical students’ learning outcome about approach to coma. BMC Med Educ. 2021;21(1):347.

17. Park SK, Kim HJ. Development and Evaluation of Virtual Reality-based Simulation Content for Nursing Students Regarding Emergency Triage. J Korean Acad Fundam Nurs. 2023;30(2):292–301.

18. Perron JE, Coffey MJ, Lovell-Simons A, Dominguez L, King ME, Ooi CY. Resuscitating cardiopulmonary resuscitation training in a virtual reality: Prospective interventional study. J Med Internet Res. 2021;23(7).

19. Siah RCJ, Xu P, Teh CL, Kow AWC. Evaluation of nursing students’ efficacy, attitude, and confidence level in a perioperative setting using virtual‐reality simulation. Nurs Forum (Auckl). 2022;57(6):1249–57.

20. Smith S, Farra S, Hodgson E. Evaluation of two simulation methods for teaching a disaster skill. BMJ Simul Technol Enhanc Learn. 2021;7(2):92‐96.

21. Traister TAA. Virtual reality simulation’s influence on nursing students’ anxiety and communication skills with anxious patients: A pilot study. Clin Simul Nurs. 2023;82:101433.

22. Wan T, Liu K, Li B, Wang X. Effectiveness of immersive virtual reality in orthognathic surgical education: A randomized controlled trial. J Dent Educ. 2024 Jan;88(1):109–17.

23. Wilson AS, O’Connor J, Taylor L, Carruthers D. A 3D virtual reality ophthalmoscopy trainer. Clin Teach. 2017;14(6):427–31.

24. Wu ML, Chao LF, Xiao X. A pediatric seizure management virtual reality simulator for nursing students: A quasi-experimental design. Nurse Educ Today. 2022;119:105550.

25. Zackoff MW, Real FJ, Sahay RD, Fei L, Guiot A, Lehmann C, et al. Impact of an immersive virtual reality curriculum on medical students’ clinical assessment of infants with respiratory distress. Pediatr Crit Care Med. 2020;21(5):477–85.

26. Zackoff MW, Young D, Sahay RD, Fei L, Real FJ, Guiot A, et al. Establishing objective measures of clinical competence in undergraduate medical education through immersive virtual reality. Acad Pediatr. 2021;21(3):575–9.
