# Supplementary material for: Virtual reality for assessment in undergraduate nursing and medical education – a systematic review
Source: BMC Med Educ. 2025 Feb 22;25:292. doi: 10.1186/s12909-025-06867-8 (PMC11846274; doi:10.1186/s12909-025-06867-8)
Supplement: Supplementary file 3 — Supplementary Material 3: Additional File 3: MERSQI Scores for each study. [file 12909_2025_6867_MOESM3_ESM.docx]

**Additional File 2: Comprehensive justifications for study inclusion in the systematic review**

| **No** | **Title** | **Author** | **Journal** | **Year** | **Population** | **HMD type** | **VR assessment (direct quotes)** |
| --- | --- | --- | --- | --- | --- | --- | --- |
| **1** | Using Virtual Simulations to Assess Situational Awareness and Communication in Medical and Nursing Education: A Technical Feasibility Study | Steven J. Anbro, Alison J. Szarko, Ramona A. Houmanfar, Amber M. Maraccini, Laura H. Crosswell, Frederick C. Harris, Michelle Rebaleati, Luka Starmer | Journal of Organizational Behavior Management | 2020 | Medical and nursing students | HTC Vive VR Headset / Tobii eye-tracking sensors | *“All participants were audio/video recorded during their observations of the medical simulation. Video recordings were then transcribed and each of the 10 predetermined opportunities for verbal checkbacks was assessed for accuracy”*[1] |
| **2** | Teaching ultrasound-guided peripheral venous catheter placement through immersive virtual reality | Nanna L. Andersen, Rune O. Jensen, Stefan Posth, Christian B. Laursen, Rasmus Jørgensen, Ole Graumann | Medicine | 2021 | Medical students | HTC Vive | *“When satisfied with the position of the PVC, the participants would press an “evaluate position” button inside IVR and receive feedback on whether the PVC had been correctly placed before they moved on to the next scenario”*[2] |
| **3** | Virtual Simulation in Nursing Education: Headset Virtual Reality and Screen-based Virtual Simulation Offer A Comparable Experience | Sayed Azher, Amanda Cervantes, Caroline Marchionni, Keerat Grewal, Hugo Marchand, Jason M. Harley | Clinical Simulation in Nursing | 2023 | Nursing students | Oculus Quest 2 VR headsets | *“After completing the VS, students received a performance score (1%-100%). This score was calculated by the software based on the students’ behaviors within the simulation relative to perfect management of the patient.”*[3] |
| **4** | Is individual practice in an immersive and interactive virtual reality application noninferior to practicing with traditional equipment in learning systematic clinical observation? A randomized controlled trial | Helen Berg, Aslak Steinsbekk | BMC Medical Education | 2020 | Medical and nursing students | Oculus Rift 5 or Oculus Quest | *“Feedback: When the user select that all documentations are done, a scoreboard appears with detailed feedback and a summary maximum of three stars, covering order of observations, whether all observations were done and if the values from the observations were correct.”*[4] |
| **5** | The effect of self‑practicing systematic clinical observations in a multiplayer, immersive, interactive virtual reality application versus physical equipment: a randomized controlled trial | Helen Berg, Aslak Steinsbekk | Advances in Health Sciences Education | 2021 | Medical and nursing students | Oculus Rift S or Oculus Quest | *“When the user on the left side of the bed completes the ABCDE observations, feedback on performance is automatically generated and all the users automatically rotate one place to the right around the bed.”*[5] |
| **No** | **Title** | **Author** | **Journal** | **Year** | **Population** | **HMD type** | **VR assessment (direct quotes)** |
| **6** | The effects of an immersive 3d interactive video program on improving student nurses' nursing skill competence: A randomized controlled trial study | Ying-Cheng Chao, Sophia H. Hu, Hsiao-Yean Chiu, Pi-Hua Huang, Hsiu-Ting Tsai, Yeu-Hui Chuang | Nurse Education Today | 2021 | Nursing students | HTC Vive | *“they could also interact with the learning material while watching the program. It took about 10–20 min to watch the program at their own pace (the time the users answered the question and the way they interacted with the program).”*[6] |
| **7** | The effects of introducing virtual reality communication simulation in students’ learning in a fundamentals of nursing practicum: A pragmatic randomized control trials | Chia-Hui Chou, Hui-Chen Tai, Shu-Ling Chen | Nurse Education in Practice | 2023 | Nursing students | HTC Vive Focus 3 VR | *“To track the interaction frequency and learning effect of the program, the system included a scoring statistics platform to track the students’ learning performance in the virtual reality scenario.”*[7] |
| **8** | The specialty mentor effect in enhancing surgical experience of medical students: A randomised control trial* | Aoife Feeley, Iain Feeley, Matthew Lee, Khalid Merghani, Eoin Sheehan | British Journal of Surgery | 2022 | Medical students | HMD type not described, but evident through software and figure | *“Simulation-derived metrics following each module attempt by participants was collected to evaluate the impact of supervision on participant performance.”*[8] |
| **9** | Educational considerations based on medical student use of polygenic risk information and apparent race in a simulated consultation | Brittany M. Hollister, Emma M. Schopp, Sydney H. Telaak, Ashley J. Buscetta, Alexander P. Dolwick, Christopher J. Fortney, Vence L. Bonham, Susan Persky | Genetics in Medicine | 2022 | Medical students | HTC Vive Pro headset and 1 hand controller | *“Transcripts of the participants’ verbalizations during the virtual interaction were also coded by 2 coders using a closed-ended codebook”*[9] |
| **10** | Case study of virtual reality sepsis management instructional design and ITEM outcomes | Chris Jacobs, Kriti Vaidya, Lauren Medwell, Tim Old, Richard Joiner | Journal of Visual Communication in Medicine | 2023 | Medical students | Quest 2 HMD with 2 controllers | *“On completion of the simulation the application provided tailored feed-back to indicate missed points on correct management.”*[10] |
| **11** | Using Virtual Reality Head-Mounted Displays to Assess Skills in Emergency Medicine: Validity Study | Marie Høxbro Knudsen, Niklas Breindahl, Tor-Salve Dalsgaard, Dan Isbye, Anne Grethe Mølbak, Gerhard Tiwald, Morten Bo Søndergaard Svendsen, Lars Konge, Joanna Bergström, and Tobias Todsen | Journal of Medical Internet Research | 2023 | Medical students | Oculus Quest 1 and 2 | *“The participants chose their answers with controllers shown as 2 hands in the HMD. Following each MCQ, the correct answer was revealed as a narrator’s voice supervising the junior doctor, and the scenario continued independent of the chosen answer”*[11] |
| **No** | **Title** | **Author** | **Journal** | **Year** | **Population** | **HMD type** | **VR assessment (direct quotes)** |
| **12** | Design and Evaluation of Using Head-Mounted Virtual Reality for Learning Clinical Procedures: Mixed Methods Study | Siew Tiang Lau, Rosalind Chiew Jiat Siah, Khairul Dzakirin Bin Rusli, Wen Liang Loh, John Yin Gwee Yap, Emily Ang, Fui Ping Lim, Sok Ying Liaw | JMIR Serious Games | 2023 | Nursing student | Oculus Quest (with 2 hand-held controllers) | *“Reward systems were incorporated, with a maximum of 3 stars when the learner completed the procedures correctly. Deliberate practice and timely feedback on the learner’s performance were integrated into the case scenarios and situations to develop critical thinking and problem-solving abilities. Each IVR experience lasted 15-20 minutes to ensure no learner fatigue”*[12] |
| **13** | Usability of mental illness simulation involving scenarios with patients with schizophrenia via immersive virtual reality: A mixed methods study | Youngho Lee, Sun Kyung Kim, Mi-Ran Eom | PLOS ONE | 2020 | Nursing student | Oculus Go | *“at the end of each scenario, communication-related tasks were given, showing suggestions to make the best responses following the patients’ last dialogue which required therapeutic communication with health professionals.”*[13] |
| **14** | Dose–response of virtual reality training of paediatric emergencies in a randomised simulation-based setting | Andrea Lietz, Julian Kraller, Alexander Hoffelner, Valentin Ritschl, Angelika Berger, Michael Wagner | Acta Paediatrica | 2023 | Medical students | Oculus Quest 2 | *“Subsequently, the participants received a performance summary including feedback pertaining to potential improvements and suboptimal interventions using the VR headset”*[14] |
| **15** | Virtual Reality for Emergency MedicineTraining in Medical School: Prospective, Large-Cohort Implementation Study | Moritz Mahling, Robert Wunderlich, Daniel Steiner, Eleonora Gorgati, Teresa Festl-Wietek, Anne Herrmann-Werner | Journal of Medical Internet Research | 2023 | Medical students | Oculus Rift S | *“Afterward, students received the automatically generated results regarding their performance. Subsequently, they were required to undergo an assessment scenario on CPR that took place in the VR environment”*[15] |
| **16** | Comparison of the effectiveness of lecture instruction and virtual reality-based serious gaming instruction on the medical students’ learning outcome about approach to coma | Meysam Siyah Mansoory, Mohammad Rasool Khazaei, Seyyed Mohsen Azizi and Elham Niromand | BMC Medical Education | 2021 | Medical students | HMD type not described, but evident through text and figure 2 | *“why we focused on this feature of the game and increased the amount and speed of interaction.” // “During the game stages, the student could make only three mistakes. If a student successfully completed all the stages of the game, they would receive a reward through the game.”*[16] |

| **No** | **Title** | **Author** | **Journal** | **Year** | **Population** | **HMD type** | **VR assessment (direct quotes)** |
| --- | --- | --- | --- | --- | --- | --- | --- |
| **17** | Development and Evaluation of Virtual Reality-based Simulation Content for Nursing Students Regarding Emergency Triage | Sook Kyoung Park, Hyuk Joon Kim | Korean Academy of Fundamentals of Nursing | 2023 | Nursing students | “*study participants used the HMD to*“ [17] | *“The study participants were conducted one at a time, after the situation ended, study participants checked the evaluation results to see if casualty classifications were performed correctly.”*[17] |
| **18** | Resuscitating cardiopulmonary resuscitation training in a vitual reality: Prospective interventional study | Janaya Elizabeth Perron, Michael Jonathon Coffey, Andrew Lovell Simons, Luis Dominguez, Mark E King, Chee Y Ooi | Journal of Medical Internet Research | 2021 | Medical students | Oculus VR | *“For successful completion of the case, all steps must have been completed correctly within 10 minutes.”*[18] |
| **19** | Evaluation of nursing students' efficacy, attitude, and confidence level in a perioperative setting using virtual‐reality simulation | Rosalind C. J. Siah, Ping Xu, Cheang L. Teh, Alfred W. C. Kow | Nursing forum | 2022 | Nursing students | *“equipped with virtual‐*  *reality headsets and two touch controllers“* [19] | *“The third feature was a quiz to assess their knowledge and the effectiveness of the virtual‐reality simulation where quizzes in the form of “Yes” or “No” and “rearrangement of sequences” were added into the simulation setting for participant interaction.”*[19] |
| **20** | Evaluation of two simulation methods for teaching a disaster skill | Sherrill Smith, Sharon L Farra, Eric Hodgson | BMJ Simulation and Technology Enhanced Learning | 2021 | Nursing students | Oculus Rift CV1, controllers | *“Multiple choice questions were interspersed throughout the individual tasks to assess the trainee’s knowledge and to redirect any incorrect answers”*[20] |
| **21** | Virtual Reality Simulation’s Influence on Nursing Students’ Anxiety and Communication Skills With Anxious Patients: A Pilot Study | Tanae Alicia-Adams Traister | Clinical Simulation in Nursing | 2023 | Nursing students | HMD type not described, but evident through software and text | *“The performance analytics dashboard uses algorithms to evaluate the participants’ communication skills, issuing a percentage score ranging from 0% to 100% (Oxford Medical Simulation, 2022)”*[21] |
| **22** | Effectiveness of immersive virtual reality in orthognathic surgical education: A randomized controlled trial | Teng Wan, Kai Liu, Biao Li, Xudong Wang | The voice of dental education | 2023 | Medical students | HTC Vive Pro | *“assessment mode to evaluate operator performance in the iVR training system.[…] Four outcomes were measured[…]number of timeouts, number of instrument selection errors, number of positional and angular errors, and number of prompts required[…]A score was then calculated based on the four measured outcomes, with a maximum possible score of 100 points.”*[22] |
| **No** | **Title** | **Author** | **Journal** | **Year** | **Population** | **HMD type** | **VR assessment (direct quotes)** |
| **23** | A 3D virtual reality ophthalmoscopy trainer | Andrew S Wilson, Jake O’ Connor, Lewis Taylor, David Carruthers | The clinical teacher | 2017 | Medical students | Google Cardboard version 1 and RITECH II | *“In the quiz section the user has to correctly answer eight questions, which are subsequently scored relative to the time taken to answer them.”*[23] |
| **24** | A pediatric seizure management virtual reality simulator for nursing students: A quasi-experimental design | Mei-Ling Wu, Li-Fen Chao, Xaviera Xiao | Nurse Education Today | 2022 | Nursing students | Oculus Rift S | *“all tasks are done correctly and within 5 min, students score 100 points. After completing the simulation, an automated feedback report appears on the screen”*[24] |
| **25** | Impact of an Immersive Virtual Reality Curriculum on Medical Students’ Clinical Assessment of Infants With Respiratory Distress | Matthew W. Zackoff, Francis J. Real, Rashmi D. Sahay, Lin Fei, Amy Guiot, Corinne Lehmann, Ken Tegtmeyer, Melissa Klein | Pediatric Critical Care Medicine | 2020 | Medical students | Oculus Rift | *“Following each scenario, the student would receive feedback from the facilitator regarding his or her assessment. Standardized supplemental instruction was provided based on learner performance. Students with incorrect assessments were provided the opportunity to repeat the scenario following coaching.”*[25] |
| **26** | Establishing Objective Measures of Clinical Competence in Undergraduate Medical Education Through Immersive Virtual Reality | Matthew W. Zackoff, Daniel Young, Rashmi D. Sahay, Lin Fei, Francis J. Real, Amy Guiot, Corinne Lehmann, Melissa Klein | Academic Pediatrics | 2021 | Medical students | Oculus Rift | *“VR sessions were video recorded, deidentified.”// “Two physicians […] performed a blinded independent review of each student’s video session and provided the global assessment of performance”*[26] |

*Note*. 3D: Three-Dimensional; ABCDE: Airway, Breathing, Circulation, Disability, Exposure; HMD: Head-Mounted Display; IVR: Immersive Virtual Reality; MCQ: Multiple-Choice Question; PVC: Peripheral Venous Catheter; VR: Virtual Reality; VS: Virtual Simulation.

**References**

1. Anbro SJ, Szarko AJ, Houmanfar RA, Maraccini AM, Crosswell LH, Harris FC, et al. Using virtual simulations to assess situational awareness and communication in medical and nursing education: A technical feasibility study. J Organ Behav Manag. 2020;(1–2):129–39.

2. Andersen NL, Jensen RO, Posth S, Laursen CB, Jørgensen R, Graumann O. Teaching ultrasound-guided peripheral venous catheter placement through immersive virtual reality: An explorative pilot study. Medicine (Baltimore). 2021;100(27):1–7.

3. Azher S, Cervantes A, Marchionni C, Grewal K, Marchand H, Harley JM. Virtual simulation in nursing education: Headset virtual reality and screen-based virtual simulation offer a comparable experience. Clin Simul Nurs. 2023;79:61–74.

4. Berg H, Steinsbekk A. Is individual practice in an immersive and interactive virtual reality application non-inferior to practicing with traditional equipment in learning systematic clinical observation? A randomized controlled trial. BMC Med Educ. 2020;20(1):123.

5. Berg H, Steinsbekk A. The effect of self-practicing systematic clinical observations in a multiplayer, immersive, interactive virtual reality application versus physical equipment: a randomized controlled trial. Adv Health Sci Educ. 2021;26(2):667–82.

6. Chao YC, Hu SH, Chiu HY, Huang PH, Tsai HT, Chuang YH. The effects of an immersive 3d interactive video program on improving student nurses’ nursing skill competence: A randomized controlled trial study. Nurse Educ Today. 2021;103:104979.

7. Chou CH, Tai HC, Chen SL. The effects of introducing virtual reality communication simulation in students’ learning in a fundamentals of nursing practicum: A pragmatic randomized control trials. Nurse Educ Pract. 2023;74:103837.

8. Feeley A, Feeley I, Lee M, Merghani K, Sheehan E. The specialty mentor effect in enhancing surgical experience of medical students: A randomised control trial. Surg Elsevier Sci. 2022;20(6):383–8.

9. Hollister B, Schopp E, Telaak S, Buscetta A, Dolwick A, Fortney C, et al. Educational considerations based on medical student use of polygenic risk information and apparent race in a simulated consultation. Genet Med. 2022;24(11):2389‐2398.

10. Jacobs C, Vaidya K, Medwell L, Old T, Joiner R. Case study of virtual reality sepsis management- instructional design and ITEM outcomes. J Vis Commun Med. 2023;1–10.

11. Knudsen MH, Breindahl N, Dalsgaard TS, Isbye D, Mølbak AG, Tiwald G, et al. Using virtual reality head-mounted displays to assess skills in emergency medicine: Validity study. J Med Internet Res. 2023;25.

12. Lau ST, Siah RCJ, Dzakirin Bin Rusli K, Loh WL, Yap JYG, Ang E, et al. Design and evaluation of using head-mounted virtual reality for learning clinical procedures: Mixed methods study. JMIR Serious Games. 2023;11.

13. Lee Y, Kim SK, Eom MR. Usability of mental illness simulation involving scenarios with patients with schizophrenia via immersive virtual reality: A mixed methods study. PLOS ONE. 2020;15(9).

14. Lietz A, Kraller J, Hoffelner A, Ritschl V, Berger A, Wagner M. Dose–response of virtual reality training of paediatric emergencies in a randomised simulation‐based setting. Acta Paediatr. 2023;112(9).

15. Mahling M, Wunderlich R, Steiner D, Gorgati E, Festl-Wietek T, Herrmann-Werner A. Virtual reality for emergency medicine training in medical school: Prospective, large-cohort implementation study. J Med Internet Res. 2023;25.

16. Mansoory M, Khazaei M, Azizi S, Niromand E. Comparison of the effectiveness of lecture instruction and virtual reality-based serious gaming instruction on the medical students’ learning outcome about approach to coma. BMC Med Educ. 2021;21(1):347.

17. Park SK, Kim HJ. Development and Evaluation of Virtual Reality-based Simulation Content for Nursing Students Regarding Emergency Triage. J Korean Acad Fundam Nurs. 2023;30(2):292–301.

18. Perron JE, Coffey MJ, Lovell-Simons A, Dominguez L, King ME, Ooi CY. Resuscitating cardiopulmonary resuscitation training in a virtual reality: Prospective interventional study. J Med Internet Res. 2021;23(7).

19. Siah RCJ, Xu P, Teh CL, Kow AWC. Evaluation of nursing students’ efficacy, attitude, and confidence level in a perioperative setting using virtual‐reality simulation. Nurs Forum (Auckl). 2022;57(6):1249–57.

20. Smith S, Farra S, Hodgson E. Evaluation of two simulation methods for teaching a disaster skill. BMJ Simul Technol Enhanc Learn. 2021;7(2):92‐96.

21. Traister TAA. Virtual reality simulation’s influence on nursing students’ anxiety and communication skills with anxious patients: A pilot study. Clin Simul Nurs. 2023;82:101433.

22. Wan T, Liu K, Li B, Wang X. Effectiveness of immersive virtual reality in orthognathic surgical education: A randomized controlled trial. J Dent Educ. 2024 Jan;88(1):109–17.

23. Wilson AS, O’Connor J, Taylor L, Carruthers D. A 3D virtual reality ophthalmoscopy trainer. Clin Teach. 2017;14(6):427–31.

24. Wu ML, Chao LF, Xiao X. A pediatric seizure management virtual reality simulator for nursing students: A quasi-experimental design. Nurse Educ Today. 2022;119:105550.

25. Zackoff MW, Real FJ, Sahay RD, Fei L, Guiot A, Lehmann C, et al. Impact of an immersive virtual reality curriculum on medical students’ clinical assessment of infants with respiratory distress. Pediatr Crit Care Med. 2020;21(5):477–85.

26. Zackoff MW, Young D, Sahay RD, Fei L, Real FJ, Guiot A, et al. Establishing objective measures of clinical competence in undergraduate medical education through immersive virtual reality. Acad Pediatr. 2021;21(3):575–9.
