# Supplementary material for: Virtual reality for assessment in undergraduate nursing and medical education – a systematic review
Source: BMC Med Educ. 2025 Feb 22;25:292. doi: 10.1186/s12909-025-06867-8 (PMC11846274; doi:10.1186/s12909-025-06867-8)
Supplement: Supplementary file 4 — Supplementary Material 4: Additional File 4: Overview of used hardware and software. [file 12909_2025_6867_MOESM4_ESM.docx]

| **Hardware** | **Studies** |
| --- | --- |
| Oculus (Meta Platforms, Inc., California, United States of America) | Azher et al., 2023  Berg & Steinsbekk, 2020, 2021  Jacobs et al., 2023  Knudsen et al., 2023  Lau et al., 2023  Lee et al., 2020  Lietz et al., 2023  Mahling et al., 2023  Perron et al., 2021  Smith et al., 2021  Wu et al., 2022  Zackoff et al., 2020, 2021 |
| HTC devices (HTC Corporation, Taoyuan City, Taiwan) | Anbro et al., 2020  Andersen et al., 2021  Chao et al., 2021  Chou et al., 2023  Hollister et al., 2022  Wan et al., 2023 |
| Google Cardboard 1 (Google, California, United States of America) | Wilson et al., 2017 |
| RITECH II (Zhuhai Ritech, Guangdong, China) | Wilson et al., 2017 |
|  |  |
| *Gaming Laptops:* Alienware, Dell / not reported | Andersen et al., 2021  Mahling et al., 2023  Perron et al., 2021 |
| *Eye tracker:* Tobii (Stockholm, Sweden) | Anbro et al., 2020 |
| **Software** | **Studies** |
| VR USGIVA 1.0 (VitaSim, Odense, Denmark) | Andersen et al., 2021 |
| Gogglemind Ltd (Cardiff, UK) | Knudsen et al., 2023 |
| Precision OS virtual reality platform (Vancouver, Canada) | Feeley et al., 2022 |
| Oxford Medical Simulation Ltd. (London, UK) | Azher et al., 2023  Mahling et al., 2023  Traister, 2022 |
| Vir Sam ABCDE (Trondheim, Norway) | Berg & Steinsbekk, 2020, 2021 |
|  |  |
| *Voice Recognition:* Azure Microsoft (Washington, United States) | Wu et al., 2022 |

**Additional File 4: Overview of used hardware and software**
